# Amplification of pico-scale DNA mediated by bacterial carrier DNA for small-cell-number transcription factor ChIP-seq

**DOI:** 10.1186/s12864-014-1195-4

**Published:** 2015-02-05

**Authors:** Janus S Jakobsen, Frederik O Bagger, Marie S Hasemann, Mikkel B Schuster, Anne-Katrine Frank, Johannes Waage, Kristoffer Vitting-Seerup, Bo T Porse

**Affiliations:** The Finsen Laboratory, Rigshospitalet, Faculty of Health Sciences, University of Copenhagen, Ole Maaløes Vej 5, 2200 Copenhagen, Denmark; Biotech Research and Innovation Centre (BRIC), University of Copenhagen, Ole Maaløes Vej 5, 2200 Copenhagen, Denmark; Danish Stem Cell Center (DanStem), Faculty of Health Sciences, University of Copenhagen, 3B Blegdamsvej, 2200 Copenhagen, Denmark; The Bioinformatics Centre, Department of Biology, Faculty of Natural Sciences, University of Copenhagen, Ole Maaløes Vej 5, 2200 Copenhagen, Denmark; Present address: Danish Pediatric Asthma Center, Copenhagen University Hospital Gentofte, Ledreborg Alle 34, 2820 Gentofte, Denmark

**Keywords:** ChIP-seq, Pico-scale, Histone mark, Transcription factor, Epigenetic profile

## Abstract

**Background:**

Chromatin-Immunoprecipitation coupled with deep sequencing (ChIP-seq) is used to map transcription factor occupancy and generate epigenetic profiles genome-wide. The requirement of nano-scale ChIP DNA for generation of sequencing libraries has impeded ChIP-seq on *in vivo* tissues of low cell numbers.

**Results:**

We describe a robust, simple and scalable methodology for ChIP-seq of low-abundant cell populations, verified down to 10,000 cells. By employing non-mammalian genome mapping bacterial carrier DNA during amplification, we reliably amplify down to 50 pg of ChIP DNA from transcription factor (CEBPA) and histone mark (H3K4me3) ChIP. We further demonstrate that genomic profiles are highly resilient to changes in carrier DNA to ChIP DNA ratios.

**Conclusions:**

This represents a significant advance compared to existing technologies, which involve either complex steps of pre-selection for nucleosome-containing chromatin or pre-amplification of precipitated DNA, making them prone to introduce experimental biases.

**Electronic supplementary material:**

The online version of this article (doi:10.1186/s12864-014-1195-4) contains supplementary material, which is available to authorized users.

## Background

Genomic mapping of histone modifications, their writers, readers and erasers as well as transcription factors (TFs) has become a house-hold approach to study the genome-wide regulation of gene expression programs [1,2]. The most widely applied method to generate such global mapping data is Chromatin ImmunoPrecipitation coupled with high-throughput sequencing (ChIP-seq), which however generally requires millions of cells as input material (e.g. [3]). Scarcity of cells in distinct, isolated *in vivo* populations such as phenotypically defined hematopoietic stem and progenitor cells has thus hampered direct experimentation on these. Examining such sub-populations is of intense interest for the elucidation of mechanisms governing lineage choice and commitment as well as transcriptional de-regulation in disease. Some researchers have cultured harvested cells to achieve sufficient cell numbers and performed ChIP-seq on cells undergoing differentiation *in vitro*, but this approach can give rise to biases due to culture conditions (e.g. [4]). Recent advances in the methodology have demonstrated successful ChIP-seq on very limited cell numbers [5,6]. However, these techniques are impeded by additional rounds of pre-amplification, potentially making them prone to introduce artifacts. One of these methods was established on cultured cells [6], while the other is limited to showing ChIP-seq with antibodies against histone modifications [5], yielding significantly more immunoprecipitated DNA than ChIP with antibodies to transcription factors. Furthermore, the complexity and cost of pre-amplification deters the implementation of the existing methods in many laboratories. Another recent method uses an elegant step of pre-ChIP indexing of Histone 3 containing chromatin fragments, allowing downstream distinction of input material [7]. The mixed inputs may thus function as mutual carriers during single-tube, small cell number ChIP. Importantly, the indexing step selects against nucleosome-poor genomic regions, making this approach less useful for unbiased investigation of genome-wide TF occupancy.

Here, we describe a straightforward and versatile work-flow for both transcription factor and histone mark ChIP-seq on low abundance cell populations isolated directly from the *in vivo* setting. A key element is the introduction of bacterial carrier DNA at the amplification step. This eliminates the previous need for pre-amplification and makes possible robust generation of sequencing libraries from picogram amounts of ChIP DNA.

## Results

### Histone mark ChIP-seq of hematopoietic cell populations

The scarcity of biologically relevant *in vivo* material is often barring global level investigations into normal development as well as the aberrant regulation behind cancer and other complex diseases. Of particular interest are the genome-wide binding patterns of transcription factors and the associated epigenetic profiles, which may pinpoint aberrant molecular mechanisms underlying transcriptional dysregulation and development of disease. Here, we use a standard FACS regimen (Additional file 1: Figure S1) to isolate a specific hematopoietic GMP-blast population from *Cebpa*^*p30/p30*^ mice expressing a truncated variant of the myeloid transcription factor CEBPA [8]. These mice develop acute myeloid leukemia with complete penetrance, and have been studied in detail [9-12]. However, the precise molecular dysregulation driving leukemogenesis remains obscure. We therefore developed a ChIP-seq assay compatible with the numbers of isolated leukemic cells from the *in vivo* context.

**Figure 1:**
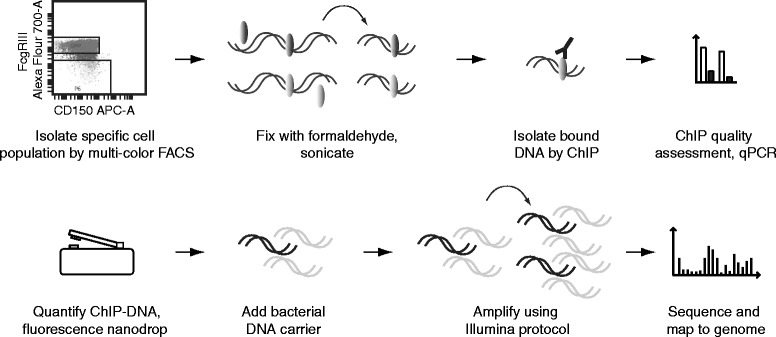
**Generation of genomic coverage maps of DNA-associated proteins from rare, isolated cell populations.** Schematic depiction of small-scale ChIP-seq workflow, including pico-gram DNA quantification and addition of fragmented bacterial DNA carrier. ChIP’ed DNA in black, carrier-DNA in light grey.

**Figure 2:**
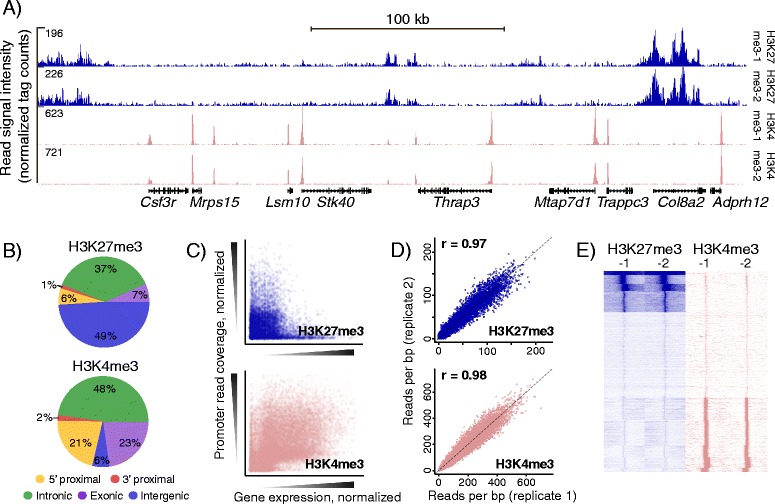
**Validation of small-scale ChIP-seq histone mark maps. (A)** Two H3K27me3 and H3K4me3 profiles and Refseq gene positions across a 400 kb region on chromosome 4. **(B)** Genomic distribution of all mapped reads, rounded numbers. **(C)** Scatterplots of promoter histone methylation and mRNA expression in GMP-phenotype blast cells. Each data point represents a single gene. Quantile normalized. **(D)** Quantitative comparisons of promoter read coverage (reads per kilobase) from two independent biological replicates of each histone modification using 1-kb TSS centered bins. Quantile normalized, 1/1 diagonal indicated by dashed line (r = pearson correlation coefficient, calculated on normalized data, 1 outlier pre-filtered). **(E)** SeqMiner cluster heatmaps showing signal intensities for both replicates of H3K27me3 and H3K4me3 in a 20000 bp region centered on Refseq TSS positions.

First, we optimized our ChIP protocol for small cell numbers, which is described in detail here for clarity. Immediately after the sorting procedure, isolated cells were exposed to formaldehyde for cross-linking chromatin-associated proteins to the DNA, washed and snap frozen in liquid nitrogen. Next, they were subjected to sonication to break the chromatin into suitably sized fragments (Figure 1 and Methods). We found that careful inspection of the DNA size distribution of each batch of chromatin was useful to prevent further processing of low quality samples. This was achieved either by processing a parallel sample of c-Kit enriched BM cells, providing a sufficient cell number for standard gel electrophoresis, or by direct inspection of each sample using the Bioanalyzer DNA1000 assay (Methods and (Additional file 2: Figure S2)). Chromatin from roughly 125,000 cells, equivalent to 250–300 ng of naked DNA, was used as input for each ChIP experiment with antibodies against the histone marks H3 Lys27 trimethylation (H3K27me3) or H3 Lys4 trimethylation (H3K4me3), performed in siliconized tubes with optimized washing conditions and titrated antibody and antibody-binding beads (Methods). Utilizing a thorough approach of extended protein degradation and de-crosslinking steps, as well as phenol-chloroform extraction for retrieving ChIP DNA ensured robust high recovery. This approach allowed us to effectively enrich for genomic sequences associated with either H3K27me3 or H3K4me3 as assessed by quantitative PCR (qPCR) (Additional file 3: Figure S3). The H3K27me3 ChIP produced ca. 2 ng of DNA for each sample. By making minor but important changes to the standard Illumina protocol, we were able to consistently amplify the 2 ng ChIP DNA to generate libraries for high-throughput sequencing (Methods). The H3K4me3 ChIP yielded an amount of DNA below the effective range of standard absorbance or fluorescence assays. We circumvented this obstacle by taking advantage of the fluorescence Nanodrop instrument, which allows reliable detection of DNA down to 5 pg/ul in a 1 ul sample volume (Additional file 4: Figure S4). With this approach, H3K4me3 ChIP DNA was measured to ca. 700 pg DNA, which we pooled to obtain the 2 ng sufficient for robust amplification (Methods). Using the Illumina Hiseq platform, we deep sequenced two libraries derived from two biologically independent samples for each of the two histone marks (Additional file 5: Table S1). We processed the aligned reads into genomic coverage profiles using standard procedures (Methods). Visual analysis of the profiles suggested a good concordance with previous findings [5,13], showing enrichment of the H3K27me3 mark in gene bodies, intergenic regions as well as promoters and H3K4me3 in gene promoter regions (Figure 2A). A quantitative analysis mapped H3K27me3 reads as 6% in promoter (5’ proximal) and 56% in gene body locations (intronic/exonic), while 21% of H3K4me3 reads resided in promoters (Figure 2B). Promoter H3K4me3 modifications were positively and H3K27me3 negatively correlated with activity of associated genes, as observed previously (e.g. [5,13-17]) (Figure 2C). Finally, we assessed the reproducibility of our ChIP-seq approach by comparing coverage in promoter regions from two biologically independent replicates and found a high degree of correspondence, both by visual inspection (example in Figure 2A and E) and quantitative measures (Figure 2D). In the examined cell type, we observe a near mutually exclusive pattern of H3K4me3 and H3K27me3 marks in promoters (Figure 2E), as opposed to e.g. embryonic stem cells displaying a set of double-marked promoters at poised genes, a hall-mark of the undifferentiated state [18,19].

### Transcription factor ChIP-seq of specific, isolated cell populations

Chromatin-associated proteins with limited genomic distributions generally yield less ChIP DNA when compared to chromatin components showing broader distributions. This is in line with our observations for H3K4me3 versus H3K27me3 modifications, with characteristic peak-like and broad distributions, respectively (e.g. [13]). Accordingly, we expected transcription factor ChIP to yield even less output DNA. To address this issue we used a larger input cell number. We performed ChIP with antibodies against CEBPA, a transcription factor known to be expressed in the GMP blast population [10], using chromatin from 250,000 or 500,000 cells, corresponding to 500–1000 ng input DNA per sample. By performing qPCR for amplicons covering known CEBPA target sequences, we verified the quality of the immuno-precipitation step (Additional file 3: Figure S3). Utilizing the fluorescent nanodrop, we measured the ChIP DNA yields of down to 250 pg (250,000 cells), an amount prohibitive for standard use as a starting point for Illumina amplification.

**Figure 3:**
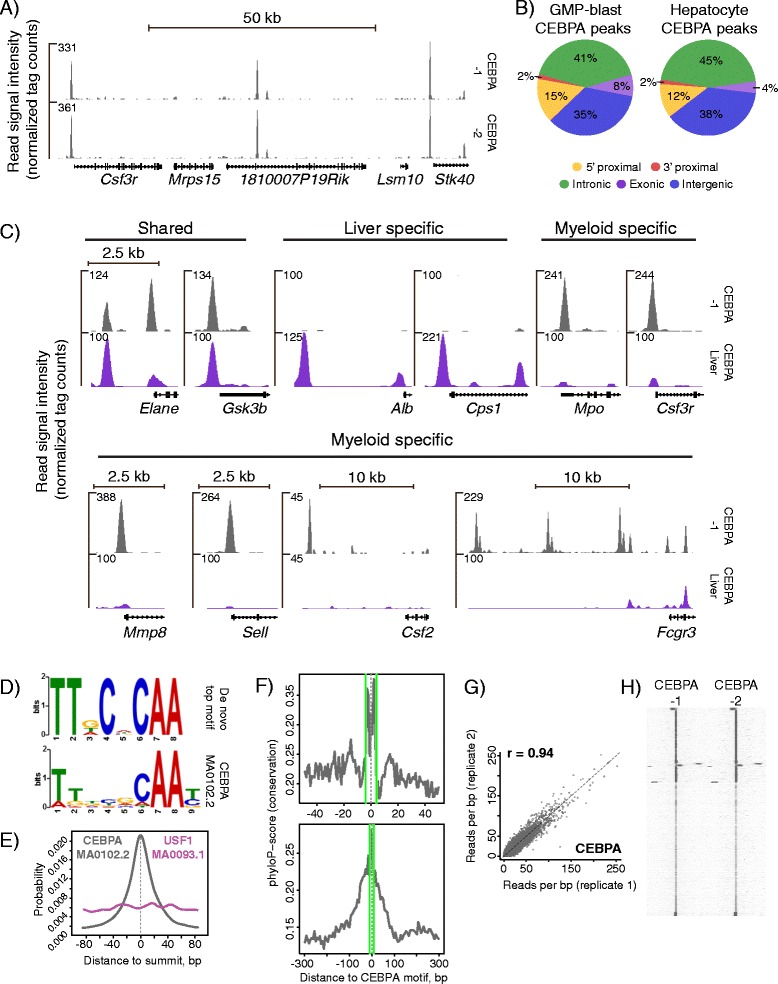
**Validation of small-scale transcription factor ChIP-seq. (A)** Profiles generated from 300 pg CEBPA ChIP-DNA. Region spanning a 100 kb region on chromosome 4. **(B)** Genomic distribution of top 26,713 CEBPA peaks (fold 30 cut-off) from GMP-blasts compared with 7,660 CEBPA peak positions in hepatocytes (fold 30 cut-off, data from [21]), rounded numbers. **(C)** CEBPA coverage tracks from GMP-blast and hepatocyte gene loci of shared, specific liver or myeloid cell expression. See text for full gene names. **(D)** De-novo motif search output from MEME (*upper panel*), and top match JASPAR motif CEBPA (MA0102.2), both in logo format (*lower panel*). Data set as in D. **(E)** Distribution of the top match motif CEBPA (MA0102.2, *grey*) in 200 bp regions centered on CEBPA-1 peak summits. USF1 (MA0093.1, *purple*) included for comparison. **(F)** Phylo-P conservation plots in 100 bp (*upper panel*) and 600 bp (*lower panel*) regions centered on CEBPA-motifs in CEBPA-1 peaks. Green lines delineate motif position. **(G)** Quantitative comparison of mean read coverage of two ChIP-seq repeats, CEBPA-1 and CEBPA-2, using CEBPA-1 peak regions defined by MACS2. Quantile normalized, 1/1 diagonal indicated by dashed line (r = pearson correlation coefficient, calculated on normalized data, 1 outlier pre-filtered). MACS2 peak boundaries for CEBPA-1 **(H)** SeqMiner cluster heatmaps showing signal intensities for CEBPA-1 and CEBPA-2 profiles in 10000 bp regions centered on peak summits. For F and G, peak set as in B.

We reasoned that an absolute loss during the amplification procedure could be ameliorated by adding a DNA carrier. First, we set out to test the use of a synthetic 120-mer oligo devoid of the terminal 3’-OH group, which would prevent ligation to the amplification adaptors and hence preclude amplification during the PCR step. This gave rise to unacceptable biases as displayed by non-linear amplification of CEBPA enriched regions (data not shown), probably due to low complexity of the DNA pool during PCR amplification. We next hypothesized that addition of complex carrier DNA that could be amplified would be ideally suited to surpass this problem. Hence, we performed an in silico mappability test of 10 million 50-bp randomly extracted *E. coli* sequences, of which 0 mapped to the mouse genome and 14457 (<0.15%) to the human counterpart (Additional file 6: Additional Methods). Adding fragmented bacterial DNA (1700 pg) to the CEBPA ChIP DNA (ca. 300 pg) produced the 2 ng established to perform robustly in amplification. By deep sequencing the resulting compound library, we were able to map up to 20% of the obtained sequences to the mouse genome (Additional file 5: Table S1). This corresponded with the ratio between ChIP DNA and bacterial carrier and typical ChIP-seq mapping frequencies. CEBPA peaks were observed in promoter proximal positions characteristic of many transcription factors (e.g. [20]) (Figure 3A). Quantitative assessment of CEBPA peak positions revealed a genomic distribution analogous to a thoroughly validated CEBPA ChIP-seq dataset obtained with liver cells (Figure 3B) [21]. Further visual inspection identified several examples of CEBPA peaks shared between hepatocyte and myeloid progenitor data sets (Figure 3C). Strikingly, the two archetypical homeostatic liver genes (*Albumin (Alb)* and *Carbamoyl-Phosphate Synthase 1 (Cps1)*) displayed CEBPA peaks in hepatocytes, but not in myeloid cells. Conversely, many genes characteristic of the myeloid lineage displayed CEBPA peaks only in the myeloid ChIP-seq dataset (e.g. *Myeloperoxidase (Mpo), Colony stimulating factor 3 receptor (Granulocyte)(Csf3r), Matrix Metallopeptidase 8 (Neutrophil Collagenase)(Mmp8), Selectin L(Sell), Colony Stimulating Factor 2 (Granulocyte-Macrophage)(Csf2), Fc Fragment of IgG, Low Affinity IIIb, Receptor (Fcgr3))* (Figure 3C). Several of these are known CEBPA targets. Two additional pieces of evidence supported the validity and specificity of our CEBPA genomic occupancy data. Firstly, the top hit of a de novo motif search in the enriched sequences matched the known CEBPA binding logo (Figure 3D), and this motif was found strongly enriched in the peak centers (Figure 3E). Secondly, robust conservation in these sequences were centered on CEBPA motifs, implying functional evolutionary constrain (Figure 3F). Visual and quantitative comparisons of coverage from two biologically independent repeats indicated a high degree of reproducibility (Figure 3A, G, H). Collectively, these data provide evidence that transcription factor ChIP-seq on small-cell-number populations is possible using bacterial DNA as a carrier during the amplification step.

### Amplification from picogram amounts of ChIP DNA

**Figure 4:**
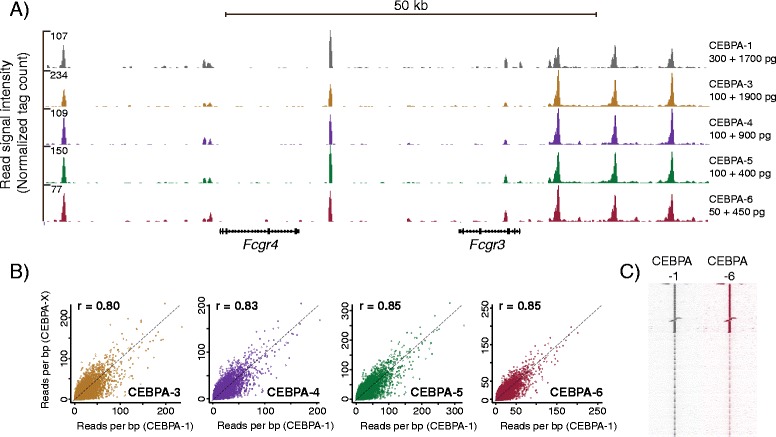
**Comparison of pico-scale transcription factor libraries. (A)** CEBPA profiles of a 80 kb region on chromosome 1, based on progressively lower amounts of input ChIP-DNA and bacterial carrier (CEBPA-1, 300 and 1700 pg; CEBPA-3, 100 and 1900 pg; CEBPA-4, 100 and 900 pg; CEBP-5, 100 and 400 pg; CEBPA-6, 50 and 450 pg). **(B)** Quantitative comparisons of mean read coverage of CEBPA-1 versus CEBPA-3 /-4/-5/-6. ChIP-seq profiles in CEBPA-1 peak regions defined by MACS2. Quantile normalized, 1/1 diagonal indicated by dashed line (r = pearson correlation, raw data). **(C)** SeqMiner cluster heatmaps showing signal intensities for profiles CEBPA-1 and CEBPA-6 at 10000 bp regions centered on peak summits. For B and C, peak set as in Figure 3B.

Many immunophenotypically defined hematopoietic compartments, e.g. the hematopoietic stem cells, consist of a very limited number of cells. ChIP from small cell populations, as has been done previously by several groups [3,5,6,22-24], consistently yield very limited quantities of ChIP DNA. Hence, we wanted to investigate if our carrier DNA amplification approach could be applied on pico-gram-scale amounts of DNA. To test this, we generated a series of compound DNA Illumina amplified libraries with varying ratios of bacterial carrier DNA and ChIP DNA from three pooled CEBPA ChIP samples (performed on 250,000 cells each) to allow direct comparisons between libraries. Aiming to minimize the dilution ratio, some of these libraries were generated using total input amounts of 1000 or 500 pg. The resulting four libraries (CEBPA-3, 100 pg ChIP DNA and 1900 pg carrier; CEBPA-4, 100 and 900 pg; CEBPA-5, 100 and 400 pg; CEBPA-6, 50 and 450 pg), all displayed amplification output yields and size distributions comparable to libraries generated from 2 ng DNA (Additional file 7: Figure S5). High-throughput sequencing of these libraries resulted in mapping frequencies close to the expected based on standard ChIP-seq mapping efficiency and ratios of ChIP DNA to bacterial carrier DNA. E.g. from a read number of roughly 85 million for CEBPA-5, 8.4 million mapped uniquely to the mouse genome (Additional file 5: Table S1). Visually, genomic coverage profiles for each of the four new libraries closely matched our previous CEBPA tracks (Figure 4A). An analysis of the degree of correlation between dilute libraries and CEBPA-1 indicated consistent, high reproducibility (Figure 4B). Strikingly, the library containing least ChIP DNA (CEBPA-6, 50 pg) display a pearson correlation of 0.85 with the 300 pg CEBPA-1 library and overall recapitulate the genomic coverage of this library (Figure 4B, C).

**Figure 5:**
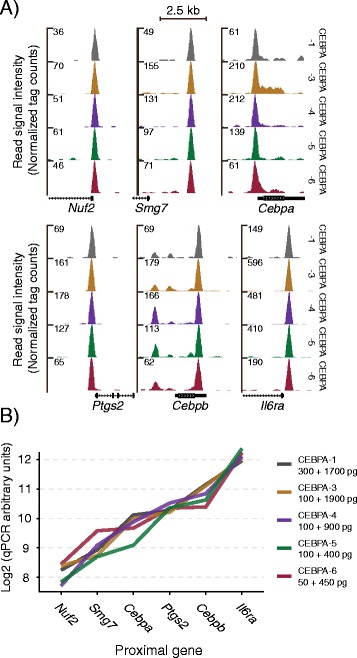
**Test of pico-scale ChIP-seq amplification linearity. (A)** CEBPA profiles of promoter regions of six known CEBP targets. **(B)** qPCR validation of linearity. Amplicons positioned in CEBPA peak regions from A. ChIP-DNA (*left*) and bacterial carrier DNA (*right*) amounts indicated.

Next, we investigated the diluted libraries for presence of CEBPA peaks at known CEBPA targets (e.g. *Nuf2, NDC80 kinetochore complex component, Smg-7 homolog, nonsense mediated mRNA decay factor, CCAAT/enhancer binding protein (C/EBP), alpha* and *- beta*, *prostaglandin-endoperoxide synthase 2* and *interleukin 6 receptor, alpha*), and found these in all coverage profiles (Figure 5A) [21]. To test our panel of libraries further, we performed qPCR to quantify amplicons corresponding to the CEBPA enriched regions mentioned above, which indicated reproducible and practically uniform amplification across dilution ratios and input amounts (Figure 5B).

### ChIP-seq using 10,000 cells

**Figure 6:**
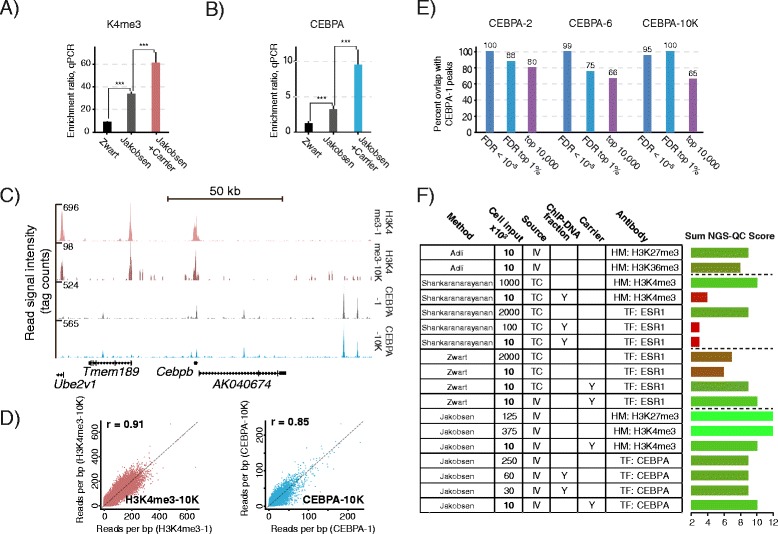
**10,000 cell histone mark and transcription factor ChIP-seq. (A and B)** ChIP using 10,000 sorted GMP-blasts as input. Our protocol (Jakobsen) with and without mRNA/Histone carrier, compared to the Zwart protocol. qPCR assessment of enrichment ratios, n = 4-6, error bars show SD. *** = P < 0.001, T-test, Holm-Sidak correction for multiple testing. **(A)** H3K4me3-ChIP, ratios are amplicon Smc4 versus the negative Sfi3. **(B)** CEBPA-ChIP, ratios are amplicon Ptgs2 versus the negative Sfi3. **(C)** H3K4me3 and CEBPA ChIP-seq profiles across a region spanning a 130 kb region on chromosome 2, using 375,000 (H3K4me3-1) or 10,000 (H3K4me3-10 K) cells as input (two upper tracks) and CEBPA ChIP-seq profiles of using 250,000 (CEBPA-1) or 10,000 (CEBPA-10 K) cells as input (two lower tracks). **(D)** Quantitative comparisons of mean read coverage of H3K4me3-1 versus H3K4me3-10 K (left panel) and CEBPA-1 versus CEBPA-10 K (right panel). All settings as described above. **(E)** Peak overlaps between ALPHA-1 and ALPHA-2, CEBPA-6 and CEBPA-10 K. MACS2 peaks were ranked for FDR (q-values) and the CEBPA-1 top 1% set tested against peaks scoring FDR < 10^−5^, in the FDR top 1% or in top 10,000. **(F)** Schematic bar diagram depicting summed NGS-QC (Next Generation Sequencing – Quality Check) score for each of the listed small-scale ChIP-seq data sets. Listed is number of cells used as input material, source of material (TC: tissue culture cells, IV: in vivo cells) and antibody target (HM: histone mark, TF: transcription factor). The ChIP-DNA fraction column denotes amplification of a fraction of DNA from a higher-cell-number ChIP corresponding to the indicated number of cells. The carrier column indicates use of mRNA/Histone as carrier during the ChIP procedure (Y = yes). All scores represent best replicate. NGS-QC sum score is calculated as a sum of quartile scores (best = 4, worst = 1) for read sub-sampling of 90, 70 and 50% of total mapped reads.

To investigate if our methodology allows ChIP-seq on limited cell numbers, we transplanted a new cohort of mice with the leukemic strain also used in the above and FACS-isolated batches of 10,000 GMP-blasts. We optimized the chromatin preparation procedure to ensure maximal input material for the small-cell-number ChIP (Additional file 8: Additional Protocols and Additional file 9: Figure S6). Thorough optimization of ChIP conditions produced robust enrichments for histone mark (H3K4me3) as well as TF (CEBPA) ChIP, both of which compared favorably to ChIPs done according to a previously published 10,000 cell ChIP methodology by Zwart et al. [24] (Figure 6A, B). The major advance in the previous study was addition of a combined mRNA/Histone carrier/blocker during the ChIP step. We amended our protocol to include such a carrier, resulting in even better enrichment as assessed by qPCR (Figure 6A, B). A small amount of 10,000 cell ChIP-DNA was used for examining enrichment and establishing quantity, leaving ca. 60 pg DNA. Parallel generation of sequencing libraries with and without bacterial carrier demonstrated that at the 60 pg ChIP-DNA range, carrier is required for robust production of high-quality Illumina libraries (Additional file 10: Figure S7). Both visual inspection and quantitative analyses of genomic coverage tracks from 10,000 cell ChIPs revealed good correspondence with libraries generated from biologically independent higher-cell-number ChIPs (Figure 6C, D, E). CEBPA-1 peaks were to a high degree shared with a biological repeat (CEBPA −2), the diluted library (CEBPA-6) and the low-cell-number ChIP (CEBPA-10K) (Additional file 6: Additional Methods). Importantly, the H3K4me3-10K profile generated from 60 pg ChIP-DNA, which display substantially fewer mapped reads (Additional file 5: Table S1), is highly similar to the H3K4me3-1 profile from 2 ng DNA resulting from ChIP without using bacterial carrier for amplification (Figure 6C, D).

The recent establishment of the online Next Generation Sequencing Quality Control (NGS-QC) Generator, a useful cross-platform quality assessment tool [25], allowed a quantitative comparison of our ChIP-seq approach with already published methods (Additional file 6: Additional Methods). Our methodology robustly produced NGS-QC scores matching or exceeding those from previous approaches (Figure 6F and Additional file 11: Table S2). Specifically, Shankaranarayanan (LinDA) amplification [6] resulted in considerably lower NGS-QC scores at the 10,000 cell range, while Adli amplification [5] and the Zwart methodology [24] produced data sets with comparable scores for histone modification or transcription factor ChIP-seq profiles, respectively (Figure 6F).

## Discussion and conclusions

Here, we present a complete work-flow for ChIP-seq on the limited cell populations of distinct *in vivo* cell compartments. By isolating a defined cell population with a standard immunophenotypic sorting regimen and using a rigorous and reliable ChIP approach, we were able to enrich regions marked by both broadly (H3K27me3) and more narrowly (H3K4me3) distributed histone modifications as well as the precise regions defined by occupancy of the central hematopoietic transcription factor, CEBPA. Importantly, the fluorescence nanodrop instrument allowed us to reliably measure the obtained picogram amounts of ChIP DNA. We demonstrate that these amounts of ChIP DNA could be faithfully amplified to generate libraries for Illumina sequencing by addition of fragmented *E. coli* carrier DNA. Further, we show the resulting coverage profiles to match previous data for the actively transcribed gene associated (H3K4me3) and repressed gene associated (H3K27me3) histone marks, and to specifically recover known myeloid specific CEBPA target regions. We provide evidence that CEBPA bound regions from a pool of ChIP’ed DNA down to 50 pg are amplified in a linear manner with our technique. Finally, we demonstrate that our straight-forward methodology can produce high-quality ChIP-seq coverage tracks from as little as 10,000 isolated *in vivo* cells using antibodies against histone mark (H3K4me3) as well as transcriptional factor (CEBPA) antibodies. Importantly, the H3K4me3-ChIP 10,000 cell genomic coverage profile is very similar to the profiles generated without bacterial carrier (H3K4me3-1/-2), indicating that the addition of bacterial carrier does not impede amplification or Illumina sequencing.

Our methodology can be used to elucidate important biological circuitries at a global level. For instance, we can clearly detect differences of direct targets of CEBPA in specific cell types as illustrated in Figure 3. Importantly, two recently published studies demonstrate the usefulness of our approach by revealing molecular mechanisms behind initiation of acute myeloid leukemia and regulation of hematopoietic stem cells [26,27]. Furthermore, our technology should combine easily with other genomics approaches, for instance Chia-PET [28], to permit generation of libraries from otherwise prohibitively small amounts of DNA.

Other available methodologies that allow generation of ChIP-seq data from limited amounts of input material rely on an extra amplification step, based either on custom designed random primers or T7 RNA-polymerase technology [5,6]. Even as these methods are very useful, introducing extra and complex steps in the amplification procedure will inevitably increase the risk of errors, and is costly and time-consuming. Our method is based on the conceptually simple addition of non-mapping DNA as carrier and should as such be easy to implement in any laboratory that already performs Illumina platform ChIP-seq.

A disadvantage of our approach is the added cost of sequencing *E. coli* DNA, generating data that will be discarded. This drawback increases with the ratio between bacterial and ChIP DNA, i.e. as fewer cells are used or less material recovered, for example as a result of poor antibodies or low expression of the ChIPed factor. We have tried to address this issue by demonstrating the feasibility of using just 500 picogram total input material for the amplification procedure. The steady decline of sequencing prices should also help reduce this disadvantage.

Several groups have optimized ChIP from very small cell numbers (e.g. [3]), whereas our study focusses on refining the amplification step of ChIP-seq. Some reports have shown the addition of carrier chromatin during the ChIP stage to facilitate the application on small cell numbers, but these methodologies are either not tested or incompatible with high-throughput sequencing [29]. Recently, Zwart et al. demonstrated an increase in the ChIP-seq signal to noise ratio by adding carrier consisting of RNA and histones [24]. While ChIP protocols generally include *bovine serum albumin* as a carrier (e.g. [30]), Zwart and coworkers speculate that combined oligonucleotide RNA and histones mimic chromatin better, and hence offer improved blocking of spurious binding. Both components are degraded prior to the amplification step, making the modification suitable for use in ChIP-seq. We compare a low-cell-number optimized version of our methodology and find it to surpass the Zwart approach for the tested antibodies (Figure 6A, B). Nevertheless, adopting the mRNA/histone ChIP level carrier considerably improves our protocol (Figure 6A, B), clearly demonstrating the value of the Zwart at al. contribution to the development of ChIP technology.

In summary, our study, in combination with the progress of sequencing technology, makes possible relatively straight-forward ChIP-seq even on very scarce cell numbers e.g. from stem cell populations. This opens the door for extensive genome-wide investigation of the regulatory circuitries at all differentiation levels of *in vivo* tissues.

## Methods

### Additional methods and protocols in additional files

(Additional file 6: Additional Methods and Additional file 8: Additional Protocols and Buffer Recipes).

### Mouse work

Blast-GMP populations were generated as described previously [31]. Briefly, fetal livers were isolated from E14.5 *Cebpa*^*p30/p30*^ (CD45.2) female embryos, homogenized and filtered through a 70 um filter. Each liver was transplanted into four lethally irradiated recipients (CD45.1) by tail vein injection. The recipients developed acute myeloid leukemia (AML) within 9–11 months after transplantation and sacrificed when moribund. The bone marrow (BM) was isolated and retransplanted into sub-lethally irradiated recipients (1.5 × 10^6 whole BM cells/recipient). The procedure was repeated to generate a tertiary leukemia, from which whole BM was retrieved for isolation of GMP-blasts. Genotyping was performed as previously described [10], utilizing genomic tail DNA as template. All mouse work was performed according to national and international guidelines and approved by the Danish Animal Ethical Committee under license #2012-15-2934-00725.

### Flow cytometry and purification of hematopoietic progenitor populations

BM cells were isolated and stained using the following antibodies: CD41-FITC, CD105-PE, Gr-1- PECy5, B220-PECy5, CD3-PECy5, Sca1-PerCp5.5, Ter119-PECy7, CD16/CD32- Alexa Flour 700, c-Kit APC-Alexa 750, CD45.2-Biotin, CD45.1-eFlour450 (all from eBioscience), Mac1-PECy5 (BD Biosciences), Streptavidin-QD655 (Invitrogen), CD150-APC (BioLegend), and 7-AAD (Invitrogen) as viability marker. For sorting, cells were c-Kit enriched using CD117 microbeads and MACS LS separations columns (Miltenyi Biotec) prior to staining. GMP-blasts used in this study were defined as previously described [8,10] (Additional file 1: Figure S1).

### Chromatin preparation

GMP-blasts were sorted into pre-wetted siliconized microcentrifuge tubes (Biozym, cat#1267-2970) on ice, containing 300 μl PBS and 3% Fetal Calf Serum (FCS) (Saveen & Werner, cat#FB-1090/500). Volume was adjusted to 1.4 ml with cold PBS-3% FCS and samples cross-linked in 1% formaldehyde for 10 minutes at room temperature using a rotator. Cross-linking was quenched using 0.125 M Glycine and samples washed twice in cold PBS-3% FCS using a swinging bucket centrifuge (4 minutes, 600 g) and soft spin settings to minimize material loss. Cells were lysed as described previously in 300 μl lysis buffer [21], applying up/down pipetting 10 times using a 100 ul tip low retention tip. Samples were sonicated using a Bioruptor sonicator plus for 30 cycles, 15/30 seconds on/off, high setting, and debris pelleted by centrifuging cold at 14,000 g for 10 minutes. Fragmentation of chromatin was evaluated on extracted DNA using either: 1) a c-Kit enriched 500,000 cell sample (see above) processed in parallel (one of six tubes sonicated simultaneously) and agarose gel electrophoresis or, 2) direct sample size inspection of a 20 ul aliquot using a Bioanalyzer (Agilent DNA1000 kit cat#5067-1504) (Additional file 2: Figure S2). Output quantity was determined for each sample using the Qubit instrument (Invitrogen, HS-assay cat#Q32851).

### Chromatin Immunoprecipitation

ChIP was performed essentially as described previously with ca. 125,000 cells for histone mark and ca. 250,000 cells for CEBPA ChIP [30]. Quanta of used antibodies (CEBPA, Santa Cruz sc-61, lot#J0407, 0.2 ug; H3K27me3, Cell signaling #9733S, lot#2, 1 ul; H3K4me3, Cell signaling #9751S, lot#2, 1 ul) and protein A beads (Sigma, cat#P9424 , 10 ul 50%/50% beads/RIPA-low salt (140 mM)) were optimized for low input amounts, using siliconized tubes (Biozym, cat#1267-2970). Preincubation was performed with 10 ul of bead-slurry to minimize background. Washing conditions and buffers as in [30], but with 5 minute, 500 μl washes; 2× RIPA-low salt (140 mM NaCl) and 2× RIPA-high salt (500 mM NaCl) for CEBPA ChIP and 1× RIPA-low salt and 3× RIPA-high salt for the histone mark antibodies, replacing previous RIPA buffer washes. Retrieval of immune-precipitated DNA was optimized using overnight proteinase K treatment and 6-hour 65°C de-crosslinking with phenol-chloroform (cat#9732) extraction in phaselock tubes (5-prime, cat#713-2536) to Lo-Bind tubes (Eppendorf, cat#525-0130) as described [30]. Pico-scale ChIP DNA concentrations were determined using the fluorescent Nanodrop 3300 PicoGreen assay (ThermoScientific and Invitrogen, cat#p7589) (Additional file 4: Figure S4). Quantitative PCR (qPCR) for ChIP validation was performed on a Roche Lightcycler 480 with primers amplifying known CEBPA target sequences or regions expected to be marked by the H3K4me3 or H3K27me3 histone modifications, comparing to predicted negative regions. Primers and ChIP enrichments are found in additional files (Additional file 12: Table S3 and Additional file 3: Figure S3). Full protocol and buffer recipes are included in additional files (Additional file 8: Additional Protocols and Buffer Recipes).

### Preparation of libraries from nano- and picogram input DNA

Amplification of 2 ng ChIP DNA was essentially performed as described by the manufacturer (NEB, cat#E6240S), with the use of precast 2% SYBR agarose gels (Invitrogen, cat#G5218-02) and excision of band size 175–400 bp. Key modifications consisted of a 30 minute ligation step, 30 minute gel solubilization at 37°C of excised gel fragments, and a prolonged, double run-through elution step (each three minutes) with preheated (55°C) elution buffer for all column purifications (Qiagen, cats#28104,28704,28004) to ensure robust recovery. Amplification of picogram precipitated DNA was performed by adding carrier up to a total of 500 pg, 1000 pg or 2 ng as indicated using purified, chromosomal *E. coli* DNA, sonicated to a size distribution of 200–500 bp (Diagenode current protocols). All steps were performed in Lo-Bind tubes (Eppendorf, cat#525-0130). Libraries were generated for duplex or triplex sequencing using a NEB kit (cat#E7335S), and size distributions assessed by Bioanalyzer (Agilent, High Sensitivity kit, cat#5067-4626), (Additional file 7: Figure S5 and Additional file 10: Figure S7). Full protocol included in additional files (Additional file 8: Additional Protocols and Buffer Recipes).

### Sequencing and mapping

All libraries were single-end sequenced on the Illumina HiSeq2000 platform at the Danish National High-throughput DNA Sequencing Centre. The resulting 50-mer reads were mapped to the NCBI7/mm9 (*Mus musculus*) genome assembly using Bowtie v. 0.12.8 with standard settings for unique mapping [32]. An overview of sequencing and mapping statistics is presented in (Additional file 5: Table S1). See additional files for mapping of bacterial carrier sequences (Additional file 6: Additional Methods).

### Visualization, statistical analysis and validation of profiles

Bigwig files for UCSC genome coverage visualization were generated using Samtools [33,34], Bedtools [35], and UCSC wigtobigwig [36]. All coverage heatmaps were built using SeqMiner [37]. Histone mark profiles were examined at transcription start site (TSSs) regions (1000 bp) based on RefSeq longest isoform gene positions [38], excluding noncoding and mitochondrial genes. Peakcalling was performed using MACS2, with parameter “--to-large” [39]. A hematopoietic progenitor IgG mock ChIP-seq data set of 31,088,767 mapped reads was used as reference [27]. CEBPA-1 peak regions for analysis were defined by MACS2 cut-off of fold 30, q-value 50. For comparisons, CEBPA-1 and −2/-3/-4/-5/-6 coverages were normalized to mapped read depth. De novo motif search was performed with MEME-ChIP online using CEBPA-1 200 bp peak regions centered on summits [40]. Centrimo was used for distribution analysis of enriched motifs [41]. See additional files for genomic position annotations of reads and peaks, ChIP track coverage/gene expression correlations and CEBPA-1 peak region Phylo-P conservation analysis (Additional file 6: Additional Methods).

### Availability of supporting data

Supporting data is included in Additional files 1, 2, 3, 4, 5, 6, 7, 8, 9, 10, 11, 12 and 13 and at NCBI Gene Expression Omnibus (GEO), entry: GSE55850.

## Additional files

Additional file 1: Figure S1. Schematic representation of fluorescence activated cell sorting strategy. This is a depiction of the FACS gating regimen used. Detailed description is provided within the file. Additional file 2: Figure S2. Validation of sonicated input chromatin. A demonstration of gel-electroforetic and Bioanalyzer analyses of input chromatin DNA size distribution. Detailed description is provided within the file. Additional file 3: Figure S3. Assessment of individual sample ChIP enrichment. This is a figure showing qPCR validation of ChIP enrichments prior to library generation. Detailed description is provided within the file. Additional file 4: Figure S4. Measuring picograms of DNA with the fluorescence Nanodrop 3300. A figure depicting fluorescent nanodrop measurement linearity and reproducibility in the 5–400 pg range. Detailed description is provided within the file. Additional file 5: Table S1. Sequencing and mapping statistics. Detailed description is provided within the file. Additional file 6:
**Additional methods.** This document is a description of additional methods including mapping of bacterial carrier DNA, genomic annotation of peaks/mapped reads, promoter coverage correlation with gene expression and conservation analysis. Additional file 7: Figure S5. Size distributions of amplified ChIP-DNA libraries. Libraries assessed by Bioanalyzer analysis. Detailed description is provided within the file. Additional file 8:
**Additional protocols.** This contains detailed protocols for small-scale ChIP, amplification methodology and buffer recipes. Additional file 9: Figure S6. Validation of sonicated input chromatin size distribution from limited cell numbers. Detailed description is provided within the file. Additional file 10: Figure S7. Illumina sequencing libraries done with or without bacterial carrier. Detailed description is provided within the file. Additional file 11: Table S2. List of comparable low-cell number datasets with NGS-QC scores. Detailed description is provided within the file. Additional file 12: Table S3. List of primer sets used. Sequences of all primers used for this study. Additional file 13:
**Additional references.** This is a list of references used in additional methods and protocols.

## Abbreviations

ChIP-seq: Chromatin ImmunoPrecipitation with deep sequencing
CEBPA: CCAAT/enhancer binding protein (C/EBP), alpha, aka C/EBPα
H3K4me3: H3K27me3, Histone modifications H3 Lysine4-trimethyl and H3 Lysine27-trimethyl
MACS: Model-based analysis of ChIP-Seq data
TF: Transcription factor
