# Supplementary material for: Amplification of pico-scale DNA mediated by bacterial carrier DNA for small-cell-number transcription factor ChIP-seq
Source: BMC Genomics. 2015 Feb 5;16(1):46. doi: 10.1186/s12864-014-1195-4 (PMC4328043; doi:10.1186/s12864-014-1195-4)
Supplement: Additional file 1: Figure S1. — Schematic representation of fluorescence activated cell sorting strategy. This is a depiction of the FACS gating regimen used. Detailed description is provided within the file. [file 12864_2014_1195_MOESM1_ESM.pdf]

Figure S1.

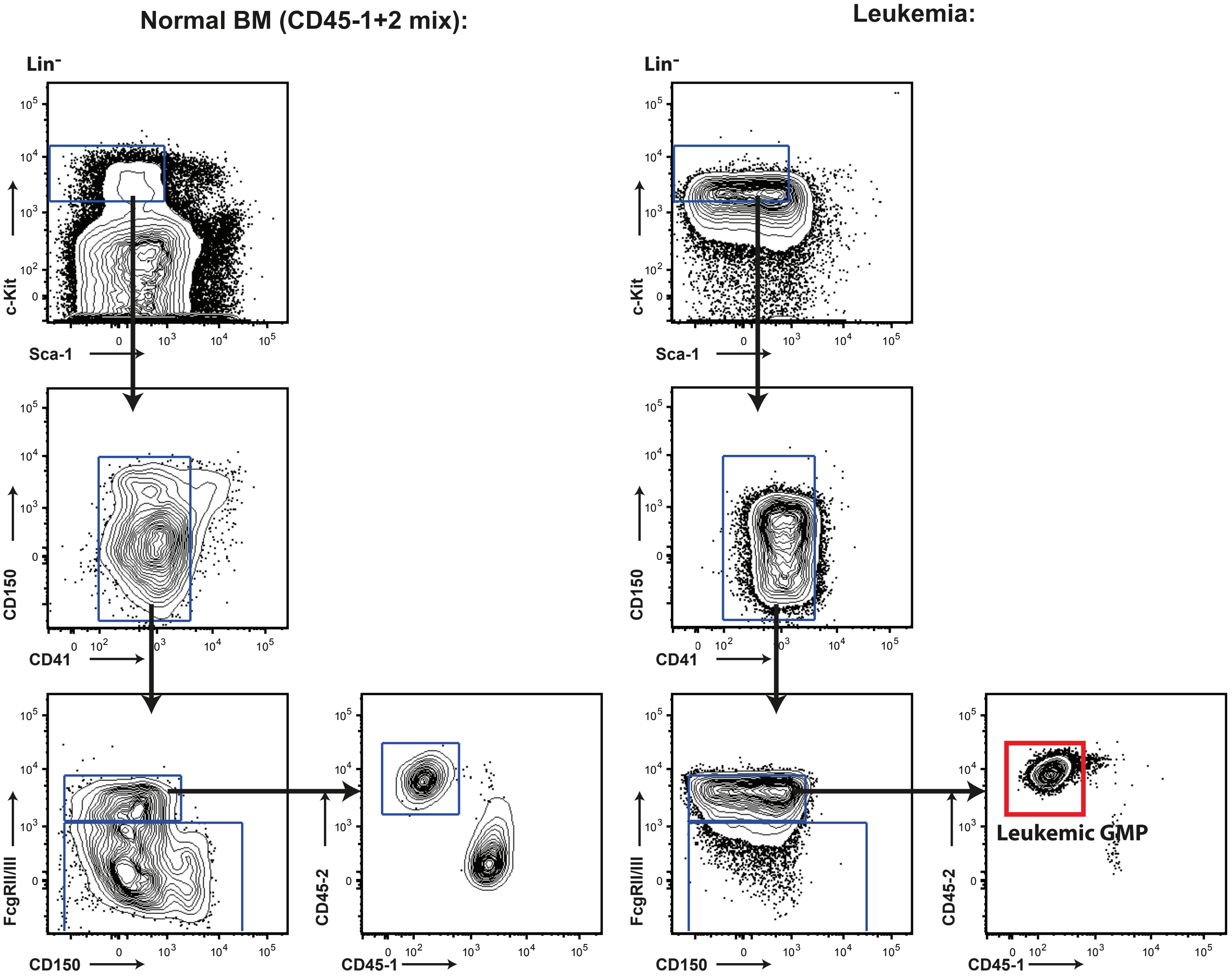

Figure S1 Schematic representation of fluorescence activated cell sorting strategy. Leukemic GMPs (right hand panels, red squares) were isolated using gates defined from normal BM populations (left hand panels, blue squares) as shown, and used for ChIP-seq experiments. Details on phenotypic markers used can be found in Materials and methods.
