# Supplementary material for: Amplification of pico-scale DNA mediated by bacterial carrier DNA for small-cell-number transcription factor ChIP-seq
Source: BMC Genomics. 2015 Feb 5;16(1):46. doi: 10.1186/s12864-014-1195-4 (PMC4328043; doi:10.1186/s12864-014-1195-4)
Supplement: Additional file 2: Figure S2. — Validation of sonicated input chromatin. A demonstration of gel-electroforetic and Bioanalyzer analyses of input chromatin DNA size distribution. Detailed description is provided within the file. [file 12864_2014_1195_MOESM2_ESM.pdf]

Figure S2.

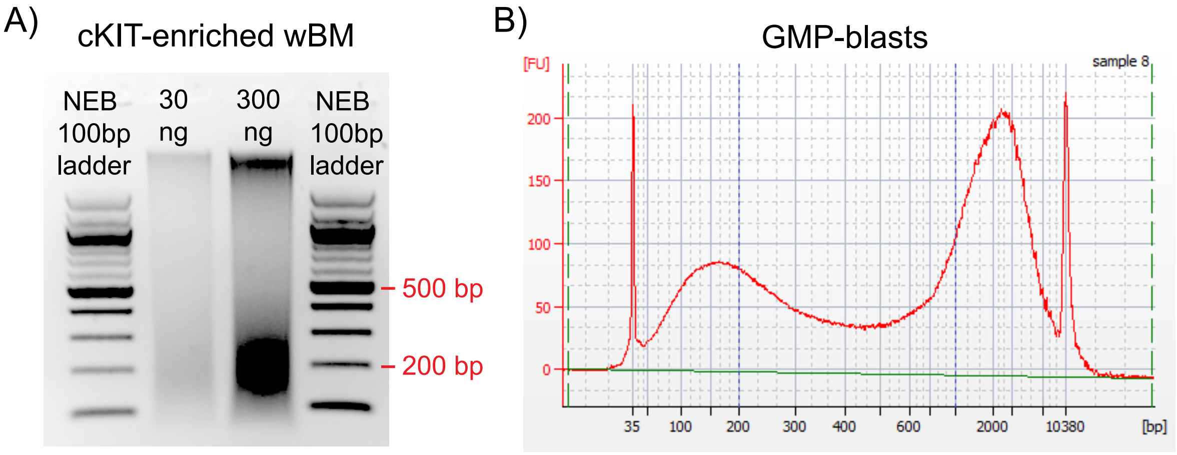

Figure S2 Validation of sonicated input chromatin. (A) Purified DNA from c-KIT enriched whole bone marrow (wBM) cells, prepared in parallel with FACS-isolated populations, subjected to standard agarose electrophoresis. (B) Purified DNA aliquot from isolated GMP-blast population chromatin assessed by Agilent Bioanalyzer High Sensitivity assay.
