# Supplementary material for: Amplification of pico-scale DNA mediated by bacterial carrier DNA for small-cell-number transcription factor ChIP-seq
Source: BMC Genomics. 2015 Feb 5;16(1):46. doi: 10.1186/s12864-014-1195-4 (PMC4328043; doi:10.1186/s12864-014-1195-4)
Supplement: Additional file 3: Figure S3. — Assessment of individual sample ChIP enrichment. This is a figure showing qPCR validation of ChIP enrichments prior to library generation. Detailed description is provided within the file. [file 12864_2014_1195_MOESM3_ESM.pdf]

Figure S3.

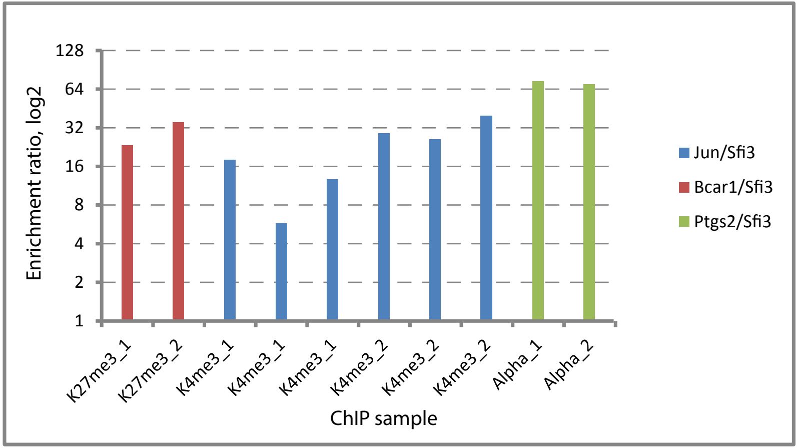

Figure S3 Assessment of individual sample ChIP enrichment. Ratio of enrichment as arbitrary qPCR units of Bcar1 (H3K27me3), Jun (H3K4me3) and Ptgs2 (CEPBA) amplicons over Sfi3 amplicons. A four-point dilution series used as standard for comparison. See supplemental Table 1 for primer sequences.
