# Supplementary material for: Amplification of pico-scale DNA mediated by bacterial carrier DNA for small-cell-number transcription factor ChIP-seq
Source: BMC Genomics. 2015 Feb 5;16(1):46. doi: 10.1186/s12864-014-1195-4 (PMC4328043; doi:10.1186/s12864-014-1195-4)
Supplement: Additional file 4: Figure S4. — Measuring picograms of DNA with the fluorescence Nanodrop 3300. A figure depicting fluorescent nanodrop measurement linearity and reproducibility in the 5–400 pg range. Detailed description is provided within the file. [file 12864_2014_1195_MOESM4_ESM.pdf]

Figure S4.

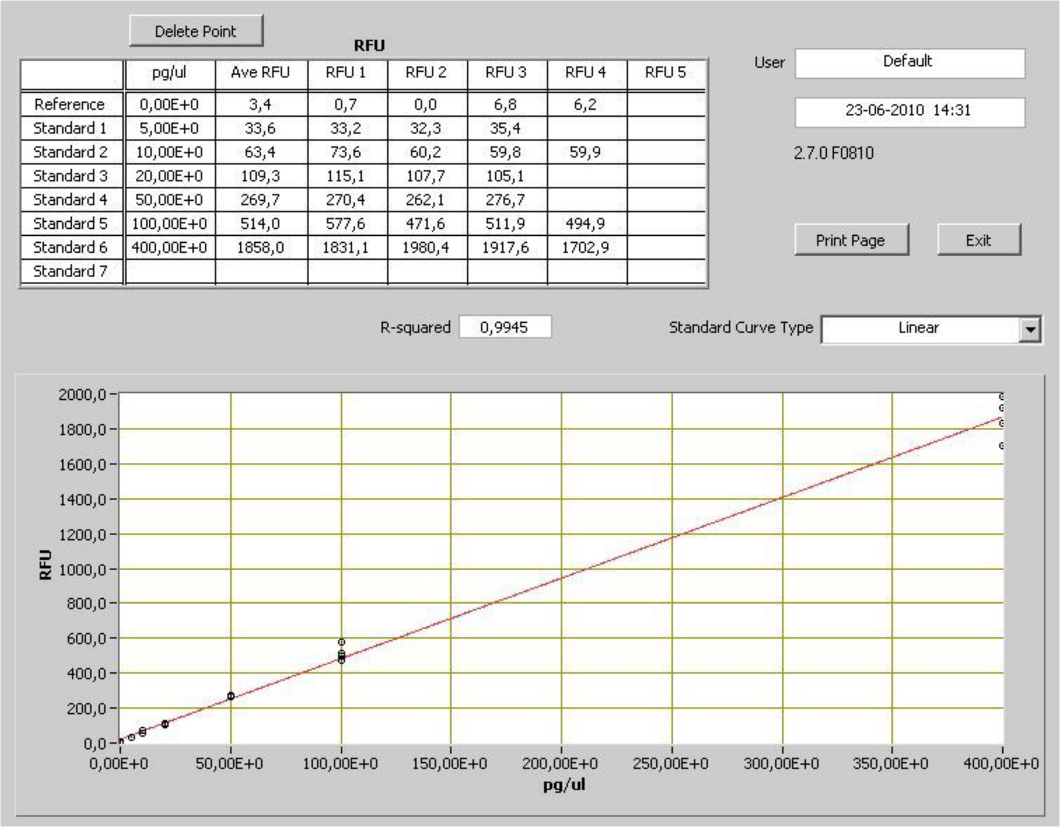

	pg/ul	Ave RFU	RFU 1	RFU 2	RFU 3	RFU 4	RFU 5
Reference	0,00E+0	3,4	0,7	0,0	6,8	6,2	
Standard 1	5,00E+0	33,6	33,2	32,3	35,4		
Standard 2	10,00E+0	63,4	73,6	60,2	59,8	59,9	
Standard 3	20,00E+0	109,3	115,1	107,7	105,1		
Standard 4	50,00E+0	269,7	270,4	262,1	276,7		
Standard 5	100,00E+0	514,0	577,6	471,6	511,9	494,9	
Standard 6	400,00E+0	1858,0	1831,1	1980,4	1917,6	1702,9	
Standard 7							

Figure S4 Measuring picograms of DNA with the fluorescence Nanodrop 3300. A seven point serial dilution of double-stranded, purified DNA (0, 5, 10, 20, 50, 100, 400 pg/µl) displaying very high linearity (r-squared = 0.9945) and measurement reproducibility, even down to 5 pg/µl.
