# Supplementary material for: Amplification of pico-scale DNA mediated by bacterial carrier DNA for small-cell-number transcription factor ChIP-seq
Source: BMC Genomics. 2015 Feb 5;16(1):46. doi: 10.1186/s12864-014-1195-4 (PMC4328043; doi:10.1186/s12864-014-1195-4)
Supplement: Additional file 6: — Additional methods. This document is a description of additional methods including mapping of bacterial carrier DNA, genomic annotation of peaks/mapped reads, promoter coverage correlation with gene expression and conservation analysis. [file 12864_2014_1195_MOESM6_ESM.pdf]

Additional File_6

<u>Additional Methods</u>

Test of mappability of bacterial carrier sequences

For testing interspecial sequence homology, 10,000,000 *E. coli* reads of 50 bp length were generated using the *E. coli* genome (strain: NC_010473), from completely random genomic loci and from both strands using R 3.1.0. Genomic sequences obtained through Bioconductor'sBSgenome package (BSgenome.Ecoli.NCBI. 20080805) [1]. All sequences containing ambiguity nucleotides were removed and unique reads were extracted. Obtained sequences were mapped to the mm9 (Mus) and hg18 (Homo) genomes, retrieved from the BS packes (BSgenome.Mmusculus.UCSC.mm9 and BSgenome.Hsapiens.UCSC. hg18) using R Biostrings v 2.32.0 package. 0 (0%) reads mapped to the mouse genome, 14457 (<0.15%) reads mapped to the human genome, excluding multimappers.

Genomic annotation of peak summits / mapped reads.

Exons, intron, 5' proximal (3000 bp upstream TSS) and 3' proximal (1000 downstream TSS) regions were acquired from UCSC using all RefSeq Gene annotation for mm9 assembly.[2, 3] Overlap was determined by bedtools intersect (with parameter -c). [4]

Correlation with gene expression

Correlation with gene expression was assessed from medians of three independent biological repeats of mRNA microarray profiling of Lp30 blast GMP population profiles from ArrayExpress ID E-MEXP-1444.[5] Raw expression intensities were RMA normalized and paired with sum of coverage in promoter regions (+/- 2000 bp around TSS) according to overlapping annotation for Affymetrix mouse430 chip and non-mitocondrial unique RefSeq Gene annotation from UCSC, respectively.

Phylo-p scores

CEBPA motif frequency matrix was obtained from the top 1000 peaks (reduced for computational reasons, but was robust even for small sets) using command line version of MEME (with parameters -nmotifs 1 -minsites 100 -minw 5 -maxw 12 -revcomp -maxsize 10000000 -dna -oc). [6] Then frequency the matrix was used with FIMO, [7] to re-find the motif in peak regions of the top 10000 peaks. Phylo P scores,[8] based on multiple alignment of 30 vertebrate genomes to the human genome was acquired from UCSC. [9] The reported scores are comprised of the mean Phylo-P score centered on the *de novo* identified CEBPA cognate motif.

Quality assessment (NGS-QC) for Chip-Seq experiments

Quality of Chip-Seq was assessed using NGS-QC (http://www.ngs-qc.org) [10]. Bam-files was used, when available, and otherwise bed-files. When raw reads was available these was mapped using bowtie2 [11] with default parameters and converted into bam-files using samtools view [12]. The tree resulting scores, either "A", "B", "C" or "D", was translated into high numbers for best scores, and mean of replicate experiments was reported when applicable. These NGS-QC scores correspond to which quartile the

experiment quality lies within, based on all publicly available data. The assessments were made ultimo august 2014.

Peak calling for assessing peak overlap

Peak calling was performed with MACS2 (version 2.0.9 20111102) [13] (using parameters -g mm --to-large). The overlap calculated on best peaks using three cutoffs: 1) Using the methodology presented by Bardet *et al.* [14] where the top 1% peaks, in terms of FDR, is compared to peaks in the other profiles either 1) being among the FDR top 1% 2) Conforming to a cut-off of FDR< 10^{-5} in each sample and 3) a fixed cut-off of 10,000 top peaks, in each sample.
