# Supplementary material for: Amplification of pico-scale DNA mediated by bacterial carrier DNA for small-cell-number transcription factor ChIP-seq
Source: BMC Genomics. 2015 Feb 5;16(1):46. doi: 10.1186/s12864-014-1195-4 (PMC4328043; doi:10.1186/s12864-014-1195-4)
Supplement: Additional file 7: Figure S5. — Size distributions of amplified ChIP-DNA libraries. Libraries assessed by Bioanalyzer analysis. Detailed description is provided within the file. [file 12864_2014_1195_MOESM7_ESM.pdf]

Figure S5.

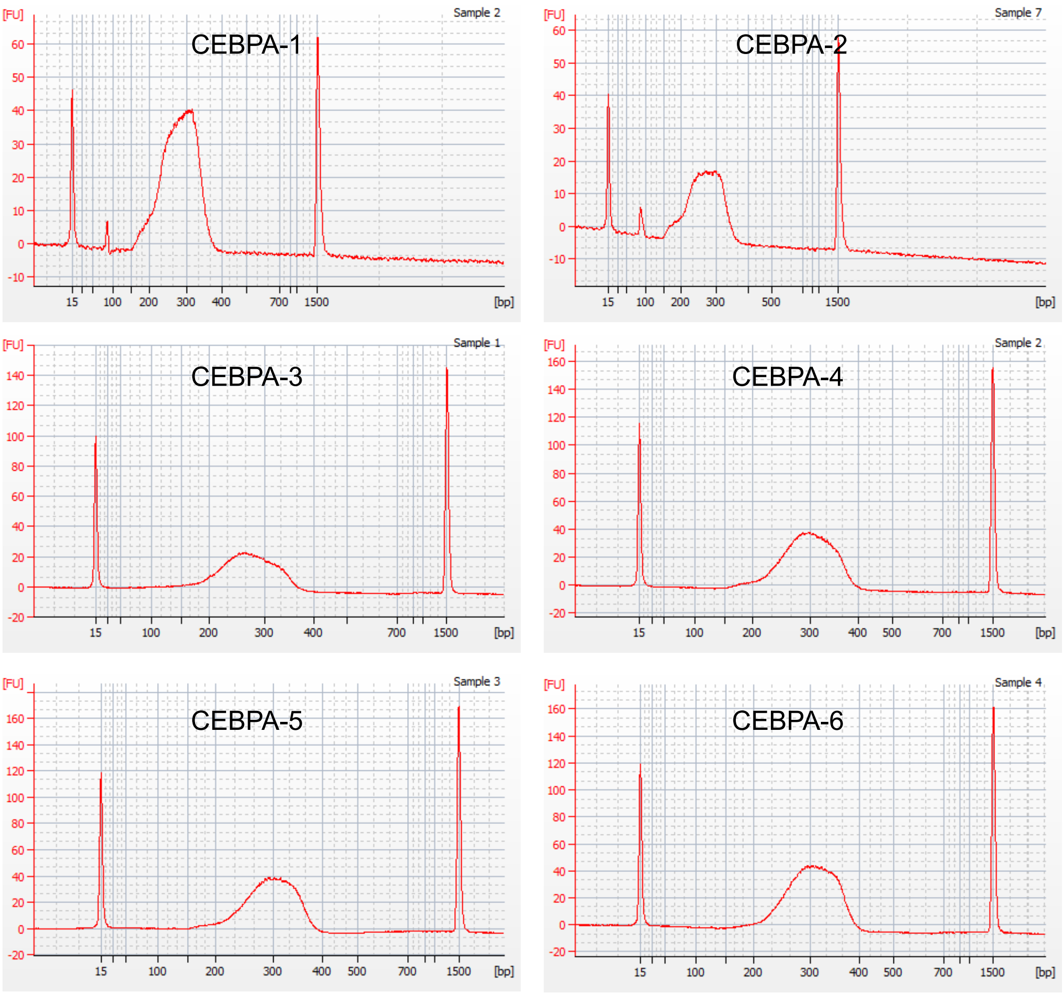

Figure S5 Size distributions of amplified ChIP-DNA libraries. 1/2 dilutions of CEBPA-1 and -2 libraries (two upper panels) and 1/5 dilutions of CEBPA-3 through -6 libraries (four lower panels) inspected by the Agilent Bioanalyzer DNA 1000 assay .
