# Supplementary material for: Amplification of pico-scale DNA mediated by bacterial carrier DNA for small-cell-number transcription factor ChIP-seq
Source: BMC Genomics. 2015 Feb 5;16(1):46. doi: 10.1186/s12864-014-1195-4 (PMC4328043; doi:10.1186/s12864-014-1195-4)
Supplement: Additional file 8: — Additional protocols. This contains detailed protocols for small-scale ChIP, amplification methodology and buffer recipes. [file 12864_2014_1195_MOESM8_ESM.pdf]

Additional File_8

Additional Protocols

Very-small-cell-number ChIP protocol (10.000 cells)

Post-sort collection and wash of cells

Cells are sorted into cold 300 µl PBS+3%FCS in low-binding siliconized tubes and kept on ice. Adjust volume to 1.5 ml with PBS+3%FCS (keep on ice!). Sorted cells can be stored on ice for a short period of time to allow simultaneous fixing of up to ca. 10 samples.

1. Add 1% formaldehyde (1% final, 40.5 µl from 37%) and Crosslink for 10 minutes at RT with rotation, fast.
2. Add 107.3 µl 2M Glycine => final conc. 0.125 M. Incubate 2 min, rotation, fast
3. (Immediately). Spin cold 4min at 600g, by placing tube in empty 15 ml tubes and swing-out centrifuge in sigma centrifuge. Wash 2x with 1 mL cold PBS +3%FCS. (Place the 1.5 ml tubes in 15 ml tubes in the large cold centrifuge – pellet will be centrifuged to the bottom of the tube)
4. Leave approximately 30 µl in the bottom, to avoid disturbing the invisible cell pellet.
5. Freeze samples at -80°C. Can be stored long-term.

Chromatin preparation and quality/quantity control

6. Resuspend thoroughly in 66 µl SDS buffer (all buffer recipes found below), always use same type of 0.5 ml microcentrifuge tubes, preferably low-binding, siliconized (Biozym cat# 710136).

2x1 µl protease inhibitors (1:1000 Leupeptin/Pepstatin =L/P and Aprotinin =A) and 10 µl (1:100) PMSF pr. ml of total lysis buffer (2/3 SDS and 1/3 Triton).

7. Incubate 20 min. R/T in a shaker, 1300 RPM,
8. Add 33 µl Triton dilution buffer to reach 100 µl, vortex (carefully), spin down cells shortly (ca. 2000 g, 10 sec.), incubate on ice 20 min. Vortex briefly and carefully, spin down shortly again
9. Sonicate 15 cycles at 30on/30off sec. at high, Diagenode Bioruptor Plus. Always use 12 tubes in diagenode 0.5 ml tube adaptor, optimize cycle number to tube plastic type. Fill empty tubes with lysis buffer to 100 µl. After 8 cycles, vortex shortly and spin down cells (ca. 2000 g, 10 sec.).
10. Take out 20 µl from each sorted sample and use as chromatin preparation control. Freeze rest of the samples in liquid nitrogen. Store samples at -80°C (long-term, >1 year storage possible).

Isolate DNA for quality (size/quantity) control, input control sample

1. Add 80 µl TE-buffer to the quality control sample. Add RNAse to 50 ug/ml (5 µl from 1 mg/ml pr. 100 µl), incubate 30min 37°C.
2. Add SDS to 0.5% (5 µl from 10% pr. 100 µl) and proteinase K to 0.5 mg/ml (2.5 µl from 20 mg/ml or 5µl from 10 mg/ml pr. 100 µl).
3. Incubate O/N at 37°C
4. Incubate at least 6hrs at 65°C.
5. Isolated DNA using Qiagen MinElute purification column system, elute in 10 µl (heated elution buffer to 55 C, run through twice, incubate 3min each time).
6. Run on an Invitrogen Qubit device for quantity and adjust to ca. 2 ng/ul for Agilent Bioanalyzer for size distribution measurements using the High Sensitivity kit.

Chromatin Immunoprecipitation

1st day: Preabsorbtion + antibody binding O/N

Preparation of beads (each IP):

1. Use 2 µl Dynabeads Protein A (Invitrogen cat# 10001D) (or Protein G) beads pr. ChIP reaction (subject to optimization, some antibodies may be best with sepharose beads, e.g. Sigma cat# P9424).
2. Wash 2x in RIPA buffer (300g, 3 min). Remove as much buffer as possible. Resuspend beads by vortexing thoroughly!

   Preclearing: wash 2 µl beads/reaction for preabsorbtion. Resuspend in 20 µl RIPA pr. final sample
3. Add RIPA up to 1000 µl to the beads used for IP and add 10 µl BSA (from 100 mg/ml)
4. Incubate IP beads O/N at 4°C rotating
5. Thaw lysates and add dialysis buffer to a total vol. of 150 µl
6. Add 120 µl incubation buffer, 3 µl PMSF and 2 x 0.3 µl protease inhibitors (L/P + A)
7. Preabsorb with beads from step 2, 1hr, 4°C, rotating
8. Spin down, move supernatant to new 1.5 ml tubes, siliconized
9. Add Antibody (approx. 0.01 µg for CEBPA and 0.2 µl H3K4me3 -cat# in methods-, subject to optimization), incubate O/N, 4°C, rotating. Optional: Add mRNA (Ambion cat# AM7846) to a total of 1 µg/ml and Histone blocker (NEB cat#M2505S) to 20 µg/ml.

2nd day: Washing and Proteinase K treatment O/N

WASHING conditions vary depending on the antiby used. *Should be optimized for each antibody*. Shown here is washing conditions for CEBPA, which are very stringent.

16. Spin down preabsorbed beads (200g, 3min), add 20µl RIPA/sample, transfer 20 µl to each IP, taking care to resuspend beads between each transfer. Use siliconized tubes.
17. Incubate 3hrs, 4°C, rotating

    Washes: All washes 500µl, at 4°C, 5min (optional: 10 min), rotation, Spin 300g, 3min between each wash. Resuspend Dynabeads thoroughly by vortexing!
18. Rinse x2 in RIPA (no washing, just spin down, add new buffer)
19. Wash x3 in RIPA
20. Wash x4 in RIPA 0.5M NaCl
21. Wash x1 in LiCl buffer
22. Wash x2 in TE
23. Resuspend in 100 µl TE add RNAse to 50 ug/ml (5 µl from 1 mg/ml pr. 100 µl), incubate 30min 37°C
24. Add SDS to 0.5% (5 µl from 10% pr. 100 µl) and proteinase K to 0.5 mg/ml (2.5 µl from 20 mg/ml or 5µl from 10 mg/ml pr. 100 µl).
25. Incubate O/N at 37°C

3rd, 4th days: De-cross-link, phenol chloroform extraction and precipitation

1. Incubate at least 6hrs at 65°C.
2. Add 100 µl TE to input samples (to a total of 200 µl)
3. Add 300 µl Phenol Chloroform and mix thoroughly, but gently
4. Add to pre-spun phase-lock tube, shake briefly, spin >15 Kg 5 min
5. Add 300 µl Chloroform, shake briefly, spin >15 Kg 5 min
9. Transfer aqueous phase (ca. 200 µl) to new 1.5 ml tube (Eppendorf LoBind)
10. Add 1 µl glycogen (5 µg/µl), 20 µl 3 M NaAc pH 5.2 and 550 ml 100% EtOH pr. 200 µl sample

(less glycogen added to allow reliable ND3300 fluorescent nanodrop quantification)
11. Incubate O/N at -80°C
12. Precipitate DNA at full speed (>20K G) 25 min, 4°C
13. Wash with 500µl 70% EtOH
14. Spin full speed, 5 min, 4°C
15. Resuspend in 20µl TE, pH 8.0

<u>Measure concentration and assess enrichment using qPCR</u>
1. Measure concentration using fluorescence Nanodrop 3300 or Qubit.
2. Asses enrichment by qPCR using a specific positive primerpair versus negative primer pair and 2 µl template in 6 µl total reactions. Negative used (Sfi3) is specific to a high-background region of the mouse chromosome 11 terminus, allowing estimation of background even in very low cell number ChIP. See Table S2 for primer sequences.

Small-cell-number ChIP protocol (>100.000 cells)

<u>Post-sort collection and wash of cells</u>
Cells are sorted into cold 300 µl PBS+3%FCS in low-binding siliconized tubes and kept on ice. For every 100.000 cells, the volume is increased by 100 µl. Up to 750,000 have been sorted and fixed by this procedure (higher cell numbers may affect fixation conditions). Adjust volume to 1.5 ml with PBS+3%FCS (keep on ice!). Sorted cells can be stored on ice for a short period of time to allow simultaneous fixing of up to ca. 10 sample.
1) Add 1% formaldehyde (1% final, 40.5 µl from 37%) and Crosslink for 10 minutes at RT with rotation, fast.
2) Add 107.3 µl 2M Glycine => final conc. 0.125 M. Incubate 2 min, rotation, fast
3) (Immediately). Spin cold 4min at 600g, by placing tube in empty 15 ml tubes and swing-out centrifuge in sigma centrifuge. Wash 2x with 1 mL cold PBS +3%FCS. (Place the 1.5 ml tubes in 15 ml tubes in the large cold centrifuge – pellet will be centrifuged to the bottom of the tube)
4) Leave approximately 30 µl in the bottom, to avoid disturbing the invisible cell pellet.
5) Freeze samples at -80°C. Can be stored long-term.

<u>Chromatin preparation and quality/quantity control</u>
Process sonication control samples and samples for CHIP in parallel:
6) Resuspend thoroughly in 0.3 ml lysis buffer, always use same type of 1.5 ml microcentrifuge tubes, preferably low-binding.
Lysis buffer (all buffer recipes found below): 2/3 SDS buffer + 1/3 Triton dilution buffer,
2x1 µl protease inhibitors (1:1000 Leupeptin/Pepstatin =L/P and Aprotinin =A) and 10 µl (1:100) PMSF pr. ml of lysis buffer
7) Incubate 20 min. R/T. (Regularly vortex)
8) Sonicate 30 cycles at 15on/30off sec. at high, Diagenode Bioruptor Plus. Always use 6 tubes, optimize cycle number to tube plastic type. Place a c-kit+ sample (3-500,000 cells) as sonication control, 1 of 6 tubes.
9) Soft spin (slow deceleration) 14.000g for 10 min at 4°C.

10) Save supernatant. Take out 20 µl from each sorted sample and use as input control. The entire c-kit+ sample is used to test sonication efficiency. Continue with the c-kit+ sample and input samples, and freeze rest of the supernatants in liquid nitrogen. Store samples at -80°C (long-term, >1 year storage possible).

Isolate DNA for quality (size/quantity) control, input and c-kit+ control sample

1. Add 90 µl TE-buffer to the input sample. Keep c-kit control sample in 300 µl. Add RNAse to 50 ug/ml (5 µl from 1 mg/ml pr. 100 µl), incubate 30min 37°C.
2. Add SDS to 0.5% (5 µl from 10% pr. 100 µl) and proteinase K to 0.5 mg/ml (2.5 µl from 20 mg/ml or 5µl from 10 mg/ml pr. 100 µl).
3. Incubate O/N at 37°C
4. Incubate at least 6hrs at 65°C.
5. Add 100 µl TE to input samples (to a total of 200 µl)
6. Add 300 µl Phenol Chloroform and mix thoroughly, but gently
7. Add to pre-spun phase-lock tube, shake briefly, spin >15 Kg 5 min
8. Add 300 µl Chloroform, shake briefly, spin >15 Kg 5 min
9. Transfer aqueous phase (ca. 200 µl for input and 300 µl for c-kit control) to new 1.5 ml tube
10. Add 10 µl glycogen (5 µg/µl), 20 µl 3 M NaAc pH 5.2 and 550 ml 100% EtOH pr. 200 µl sample (e.g. multiply the above volumes with 1.5 for the c-kit+ control)
11. Incubate O/N at -80°C (if you are in a hurry, you can do 20 min incubation for quality control sample)
12. Precipitate DNA at full speed (>20K G) 25min, 4°C
13. Wash with 500µl 70% EtOH
14. Spin full speed, 5 min, 4°C
15. Remove ethanol, dry pellet and resuspend in 20-50 µl TE. Run a 1.2% agarose gel of the c-kit+ control to assess chromatin size. Input samples can be run on an Invitrogen Qubit device for quantity and Agilent Bioanalyzer for size distribution measurements.

Chromatin Immunoprecipitation

1st day: Preabsorbtion + antibody binding O/N

Preparation of beads (each IP):

1. Use X µl (10 µl in our case, subject to optimization) Protein A (or Protein G) beads (50/50 slurry) pr. ChIP reaction
2. Wash 1x in RIPA buffer (1000g, 2 min). Remove as much buffer as possible.

Preclearing: wash 10 µl beads/reaction for preabsorbtion. Resuspend in 50 µl RIPA pr. final sample (e.g. 150 µl for 3 samples) – now ready for preclearing.

3. Add 500 µl to the beads used for IP (independent of the amount of beads) and add 10 µl BSA (from 100 mg/ml)
4. Incubate O/N at 4°C rotating

Antibody binding:

5. Thaw lysates and add dialysis buffer to a total vol. of 500 µl
6. Add 400 µl incubation buffer, 10 µl PMSF and 2x1 µl protease inhibitors (L/P + A)
7. Preabsorb with beads from step 2, 1hr, 4°C, rotating
8. Spin down, move supernatant to new 1.5 ml tubes, siliconized
9. Add Antibody (approx. 0.5-2 ug, subject to optimization), incubate O/N, 4°C, rotating

2nd day: Washing and Proteinase K treatment O/N

WASHING conditions varies depending on the antiby used. Should be optimized for each. Shown here is washing conditions for CEBPA, which are very stringent.

16. Spin down preabsorbed beads (1000g, 2min), add 50µl RIPA/sample (i.e. 150 µl for 3 samples), transfer 50 µl to each IP. Use siliconized tubes.
17. Incubate 3hrs, 4°C, rotating

Washes: All washes 500µl, at 4°C, 5min (optional: 10 min), rotation, Spin 1000xg, 2min between each wash

18. Rinse x2 in RIPA (no washing, just spin down, add new buffer)
19. Wash x1 in RIPA
20. Wash x4 in RIPA 0.5M NaCl
21. Wash x1 in LiCl buffer
22. Wash x2 in TE
23. Resuspend in 100 µl TE add RNAse to 50 ug/ml (5 µl from 1 mg/ml pr. 100 µl), incubate 30min 37°C
24. Add SDS to 0.5% (5 µl from 10% pr. 100 µl) and proteinase K to 0.5 mg/ml (2.5 µl from 20 mg/ml or 5µl from 10 mg/ml pr. 100 µl).
25. Incubate O/N at 37°C

3rd, 4th days: De-cross-link, phenol chloroform extraction and precipitation

1. Incubate at least 6hrs at 65°C.
2. Add 100 µl TE to input samples (to a total of 200 µl)
3. Add 300 µl Phenol Chloroform and mix thoroughly, but gently
4. Add to pre-spun phase-lock tube, shake briefly, spin >15 Kg 5 min
5. Add 300 µl Chloroform, shake briefly, spin >15 Kg 5 min
9. Transfer aqueous phase (ca. 200 µl) to new 1.5 ml tube (Eppendorf LoBind)
10. Add 1 µl glycogen (5 µg/µl), 20 µl 3 M NaAc pH 5.2 and 550 ml 100% EtOH pr. 200 µl sample
11. Incubate O/N at -80°C
12. Precipitate DNA at full speed (>20K G) 25 min, 4°C
13. Wash with 500µl 70% EtOH
14. Spin full speed, 5 min, 4°C
15. Resuspend in 20 µl TE, pH 8.0

<u>Measure concentration and assess enrichment using qPCR</u>

1. Measure concentration using fluorescence Nanodrop 3300 or Qubit.
2. Dilute your sample 5-10x depending on concentration before using it for qPCR.

Buffer recipes

RIPA	pH8.0	500 ml	Stock
140mM NaCl		14 ml	from 5M
10mM Tris-HCl		5 ml	from 1M
1mM EDTA		1 ml	from 0.5M
1% TritonX100		25 ml	from 20%
0.1% SDS		5 ml	from 10%
0.1% Sodium Deoxycholate		5 ml	from 10%

RIPA 0.5 M NaCl	pH8.0	1 L	Stock
500mM NaCl		100 ml	from 5M
10mM Tris-HCl		10 ml	from 1M
1mM EDTA		2 ml	from 0.5M
1% TritonX100		50 ml	from 20%
0.1% SDS		10 ml	from 10%
0.1% Sodium Deoxycholate		10 ml	from 10%

LiCl	pH8.0	500 ml	Stock
250mM LiCl		25 ml	from 5M
10mM Tris-HCl		5 ml	from 1M
1mM EDTA		1 ml	from 0.5M
0.5% Sodium Deoxycholate		25 ml	from 10%
0.5% NP-40		2.5 g	

TE	pH8.0	500 ml	Stock
10mM Tris-HCl		5 ml	from 1M
1mM EDTA		1 ml	from 0.5M

Dialysis buffer	pH8.0	100 ml	Stock
4% glycerol		4 ml	from 100%
10mM Tris-HCl		1 ml	from 1M
1mM EDTA		0.2 ml	from 0.5M
0.5mM EGTA		19 mg	

Incubation buffer	pH8.0	100 ml	Stock
0.35M NaCL		7 ml	from 5M
10mM Tris-HCl		1 ml	from 1M
2.5% TritonX100		2.5 ml	from 20%
0.25% Sodium Deoxycholate		2.5 ml	from 10%

SDS Buffer (lysis)	pH8.0	250 mL	Stock
100mM NaCl		5 ml	from 5M
50mM Tris-HCl		12.5 ml	from 1M
5mM EDTA		2.5 ml	from 0.5M
0.5% Sodium Deoxycholate		12.5 ml	from 10%

Triton Dilution Buffer	pH8.0	250 mL	Stock
100mM NaCl		5 ml	from 5M
100mM Tris-HCl		25 ml	from 1M
5mM EDTA		2.5 ml	from 0.5M
5% TritonX100		62.5 ml	from 20%

Modified Illumina amplification protocol for pico-gram-scale input DNA (Based on NEB cat# E6240L)

Starting Material: 50-200 pg of chromatin-immunoprecipitated DNA, in < 40 µl of water or elution buffer
Add sonicated bacterial carrier DNA (200-500bps) to 500 or 2000 pg.
TIPS: Use Eppendorf LoBind or siliconized tubes for all reaction steps to reduce loss. Pre-warm elution buffers to 55°C and run through twice to optimize DNA retrieval.

<u>End Repair of ChIP DNA</u>

1. In a 0.5 ml Eppendorf LoBind microfuge tube mix the following components:
   ChIP DNA: 1–40 µl
   NEBNext End Repair Reaction Buffer: 5 µl
   NEBNext End Repair Enzyme Mix: 1 µl
   Sterile H2O variable volume
   ==Total volume 50 µl
2. Incubate in a thermal cycler for 30 min at 20°C.
3. Isolate repaired DNA by Qiagen PCR purification kit, pre-warm elution buffer to 55°C, run through column twice, make sure to retrieve all elution buffer stuck in upper column compartment. Elute 44 µl.

<u>dA-Tailing of End Repaired DNA</u>

1. In a 0.5 or 1.5 ml Eppendorf LoBind microfuge tube mix the following components:
   End Repaired DNA: 44 µl
   NEBNext dA-Tailing Reaction Buffer (10X): 5 µl
   Klenow Fragment (3´ → 5´ exo –): 1 µl
   == Total volume 50 µl
2. Incubate at 37°C for 30 min.
3. Isolate dA-tailed DNA by Qiagen min-elute purification kit, pre-warm elution buffer to 55°C, run through column twice, make sure to retrieve all elution buffer stuck in upper column compartment. Elute 19 µl.

Adaptor Ligation of dA-Tailed DNA (USER step specific to multiplex adaptor oligos)

1. In a 0.5 or 1.5 ml Eppendorf LoBind microfuge tube mix the following components:
   End Repaired, dA-Tailed DNA: 19 µl
   Quick Ligation Reaction Buffer (5X): 6 µl
   Diluted NEBNext Adaptor (1.5 µM): 1 µl (NEB Singleplex or multiplex sets)
   Quick T4 DNA Ligase: 4 µl
   == Total volume 30 µl
2. Incubate at 20°C for 30 min (NB: longer than standard 15 min).
3. Add 3 µl of USER™ enzyme mix by pipetting up and down, and incubate at 37°C for 15 min.
4. Isolate ligated DNA by Qiagen min-elute purification kit, pre-warm elution buffer to 55oC, run through column twice, make sure to retrieve all elution buffer stuck in upper column compartment. Elute 20 µl.

Size selection using 2% agarose gel

1. Run electrophoresis using Life Technologies 2% EX gel and NEB 100 bp ladder as size marker. With blue light table for illumination, cut out fragment size corresponding to 175-400 bp. Use sterile, new scalpel for each sample.
2. Transfer gel fragments to 2 ml Eppendorf LoBind microfuge tubes, isolate DNA with Qiagen gel purification kits with min-elute columns. Use thermo-shaker at 37oC, 1400 RPM for 30 min to prevent dissociation of AT-rich samples. Inspect each sample carefully to ensure full resolubilization. Pre-warm elution buffer to 55°C, run through column twice, make sure to retrieve all elution buffer stuck in upper column compartment. Elute 23 µl.

PCR Enrichment of Adaptor Ligated DNA (specific for multiplexing adaptors)

1. In a 0.2 ml sterile PCR tube mix the following components:
   Adaptor ligated DNA: 23 µl
   NEBNext High-Fidelity 2X PCR Master Mix: 25 µl
   Universal PCR Primer (25 µM): 1 µl
   Index 1 Primer* (25 µM): 1 µL
   == Total volume 50 µl

 * If you are using the NEBNext Multiplex Oligos for Illumina (E#7335, #E7500), for each reaction, only one of the 12 PCR primer indices is used during the PCR step.

2. PCR cycling conditions (NB: 18 cycles, not 15 as in standard protocol):

Cycle Step	Temp	Time	Cycles
Initial Denaturation	98°C	30 sec	1
Denaturation<br>Annealing<br>Extension	98°C<br>65°C<br>72°C	10 sec<br>30 sec<br>30 sec	18
Final Extension	72°C<br>4°C	5 min<br>hold	1

3. Isolate amplified DNA by Qiagen PCR purification kit, pre-warm elution buffer to 55°C, run through column twice, make sure to retrieve all elution buffer stuck in upper column compartment. Elute 20 µl.
4. Measure DNA concentration using the Invitrogen Qubit broad range assay.
5. Dilute sample 5-10x and check library size distribution using the Agilent Bioanalyzor DNA 1000 assay.
6. Test library integrity by measuring enrichment of known target sequences by qPCR.
7. Submit to sequencing.
