# Supplementary material for: Amplification of pico-scale DNA mediated by bacterial carrier DNA for small-cell-number transcription factor ChIP-seq
Source: BMC Genomics. 2015 Feb 5;16(1):46. doi: 10.1186/s12864-014-1195-4 (PMC4328043; doi:10.1186/s12864-014-1195-4)
Supplement: Additional file 9: Figure S6. — Validation of sonicated input chromatin size distribution from limited cell numbers. Detailed description is provided within the file. [file 12864_2014_1195_MOESM9_ESM.pdf]

Figure S6.

GMP-blasts

A)

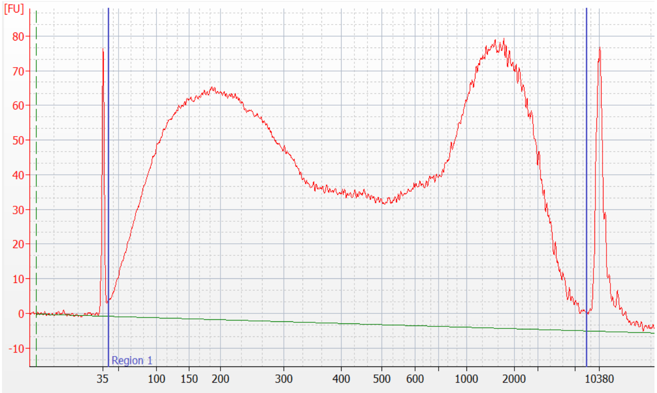

B)

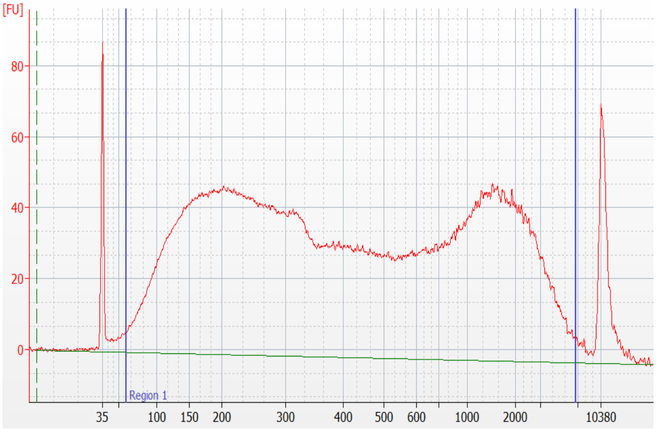

C)

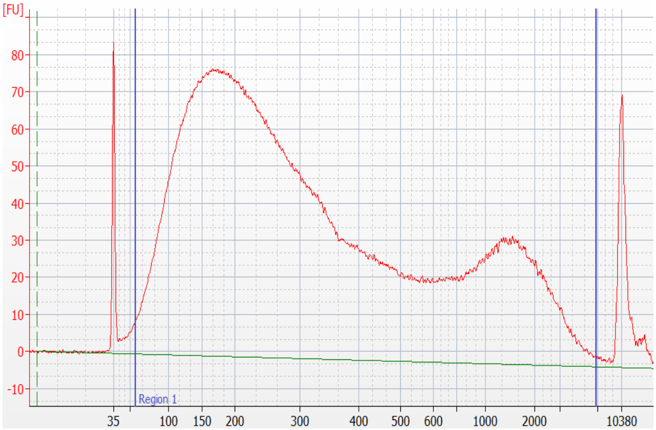

Figure S6 Validation of sonicated input chromatin size distribution from limited cell numbers. (A) 10,000 cells (B) 50,000 cells. (C) 125,000 cells. All sonicated in 0.5 ml siliconized tubes with 0.5 ml tube adaptor and resulting isolated DNA sizes inspected by the Agilent Bioanalyzer DNA high sensitivity assay (all diluted to ca. 2 ng/μl).
