# Supplementary material for: Amplification of pico-scale DNA mediated by bacterial carrier DNA for small-cell-number transcription factor ChIP-seq
Source: BMC Genomics. 2015 Feb 5;16(1):46. doi: 10.1186/s12864-014-1195-4 (PMC4328043; doi:10.1186/s12864-014-1195-4)
Supplement: Additional file 10: Figure S7. — Illumina sequencing libraries done with or without bacterial carrier. Detailed description is provided within the file. [file 12864_2014_1195_MOESM10_ESM.pdf]

Figure S7.

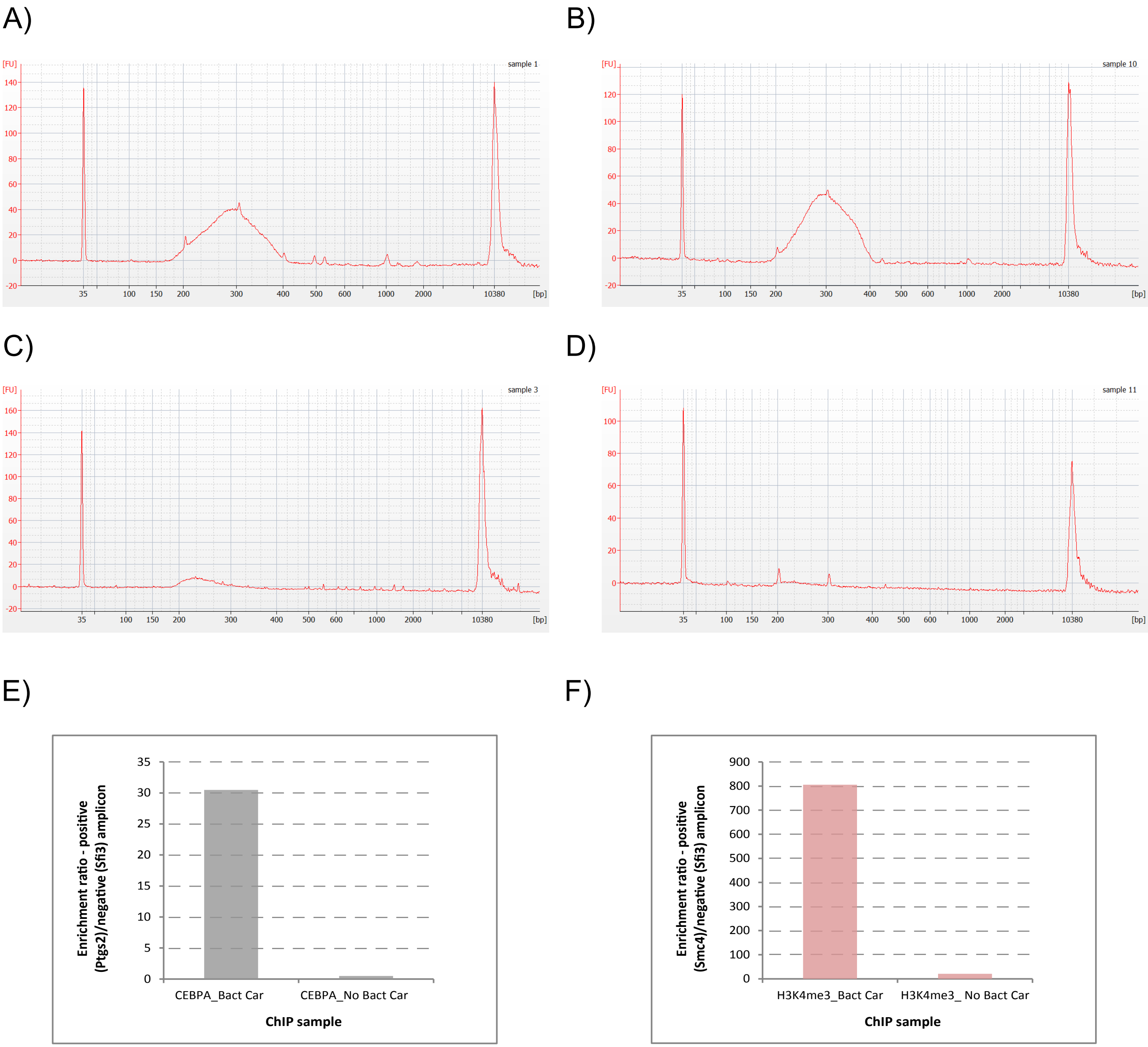

Figure S7 Illumina sequencing libraries done with or without bacterial carrier. (A) CEBPA ChIP library. (B) H3K4me3 ChIP library. A and B done with DNA from ChIP on 10,000 Cells (ca. 100 pg for CEBPA and 60 pg for H3K4me3) + 400 pg sonicated bacterial DNA carrier. (C) Failed CEBPA ChIP library (D) Failed H3K4me3 ChIP library. C and D done with DNA from ChIP on 10,000 Cells (ca. 100 pg for CEBPA and 60 pg for H3K4me3) and no bacterial DNA carrier. Output assesed by Agilent Bioanalyzer High Sensitivity assay using 1/30 dilutions. (E) Assessment of enrichment in CEBPA libraries (from A and C). Ratio of enrichment as arbitrary qPCR units of Ptgs2 over Sfi3 amplicons. (F) Assessment of enrichment in H3K4me3 libraries (from B and D). Ratio of enrichment as arbitrary qPCR units of Smc4 over Sfi3 amplicons. A four-point dilution series used as standard for comparison. See supplemental Table 1 for primer sequences.
