# Supplementary material for: Amplification of pico-scale DNA mediated by bacterial carrier DNA for small-cell-number transcription factor ChIP-seq
Source: BMC Genomics. 2015 Feb 5;16(1):46. doi: 10.1186/s12864-014-1195-4 (PMC4328043; doi:10.1186/s12864-014-1195-4)
Supplement: Additional file 13: — Additional references. This is a list of references used in additional methods and protocols. [file 12864_2014_1195_MOESM13_ESM.pdf]

Additional File_13

Additional References

1. Gentleman RC, Carey VJ, Bates DM, Bolstad B, Dettling M, Dudoit S, Ellis B, Gautier L, Ge Y, Gentry J, et al: **Bioconductor: open software development for computational biology and bioinformatics.** *Genome Biol* 2004, **5:**R80.
2. Karolchik D, Barber GP, Casper J, Clawson H, Cline MS, Diekhans M, Dreszer TR, Fujita PA, Guruvadoo L, Haeussler M, et al: **The UCSC Genome Browser database: 2014 update.** *Nucleic Acids Res* 2014, **42:**D764-770.
3. Pruitt KD, Tatusova T, Maglott DR: **NCBI Reference Sequence (RefSeq): a curated non-redundant sequence database of genomes, transcripts and proteins.** *Nucleic Acids Res* 2005, **33:**D501-504.
4. Quinlan AR, Hall IM: **BEDTools: a flexible suite of utilities for comparing genomic features.** *Bioinformatics* 2010, **26:**841-842.
5. Kirstetter P, Schuster MB, Bereshchenko O, Moore S, Dvinge H, Kurz E, Theilgaard-Monch K, Mansson R, Pedersen TA, Pabst T, et al: **Modeling of C/EBPalpha mutant acute myeloid leukemia reveals a common expression signature of committed myeloid leukemia-initiating cells.** *Cancer Cell* 2008, **13:**299-310.
6. Bailey TL, Elkan C: **Fitting a mixture model by expectation maximization to discover motifs in biopolymers.** *Proc Int Conf Intell Syst Mol Biol* 1994, **2:**28-36.
7. Grant CE, Bailey TL, Noble WS: **FIMO: scanning for occurrences of a given motif.** *Bioinformatics* 2011, **27:**1017-1018.
8. Pollard KS, Hubisz MJ, Rosenbloom KR, Siepel A: **Detection of nonneutral substitution rates on mammalian phylogenies.** *Genome Res* 2010, **20:**110-121.
9. Kent WJ, Sugnet CW, Furey TS, Roskin KM, Pringle TH, Zahler AM, Haussler D: **The human genome browser at UCSC.** *Genome Res* 2002, **12:**996-1006.
10. Mendoza-Parra MA, Van Gool W, Mohamed Saleem MA, Ceschin DG, Gronemeyer H: **A quality control system for profiles obtained by ChIP sequencing.** *Nucleic Acids Res* 2013, **41:**e196.
11. Langmead B, Salzberg SL: **Fast gapped-read alignment with Bowtie 2.** *Nat Methods* 2012, **9:**357-359.
12. Li H, Handsaker B, Wysoker A, Fennell T, Ruan J, Homer N, Marth G, Abecasis G, Durbin R, Genome Project Data Processing S: **The Sequence Alignment/Map format and SAMtools.** *Bioinformatics* 2009, **25:**2078-2079.
13. Zhang Y, Liu T, Meyer CA, Eeckhoute J, Johnson DS, Bernstein BE, Nusbaum C, Myers RM, Brown M, Li W, Liu XS: **Model-based analysis of ChIP-Seq (MACS).** *Genome Biol* 2008, **9:**R137.
14. Bardet AF, He Q, Zeitlinger J, Stark A: **A computational pipeline for comparative ChIP-seq analyses.** *Nat Protoc* 2012, **7:**45-61.
